# Noncompaction cardiomyopathy: a substrate for a thromboembolic event

**DOI:** 10.1186/1471-2261-15-7

**Published:** 2015-01-24

**Authors:** Marcelo Dantas Tavares de Melo, José Arimateia Batista de Araújo Filho, Jose Rodrigues Parga Filho, Camila Rocon de Lima, Charles Mady, Roberto Kalil-Filho, Vera Maria Cury Salemi

**Affiliations:** Heart Institute (InCor) do Hospital das Clínicas da Faculdade de Medicina da Universidade de São Paulo, São Paulo, Brazil

**Keywords:** Cardiomyopathy, Echocardiography, Magnetic resonance, Noncompaction, Thromboembolism

## Abstract

**Background:**

Noncompaction cardiomyopathy (NCC) is a rare genetic cardiomyopathy characterized by a thin, compacted epicardial layer and an extensive noncompacted endocardial layer. The clinical manifestations of this disease include ventricular arrhythmia, heart failure, and systemic thromboembolism.

**Case presentation:**

A 43-year-old male was anticoagulated by pulmonary thromboembolism for 1 year when he developed progressive dyspnea. Cardiovascular magnetic resonance imaging showed severe biventricular trabeculation with an ejection fraction of 15%, ratio of maximum noncompacted/compacted diastolic myocardial thickness of 3.2 and the presence of exuberant biventricular apical thrombus.

**Conclusion:**

Still under discussion is the issue of which patients and when they should be anticoagulated. It is generally recommended to those presenting ventricular systolic dysfunction, antecedent of systemic embolism, presence of cardiac thrombus and atrial fibrillation. In clinical practice the patients with NCC and ventricular dysfunction have been given oral anticoagulation, although there are no clinical trials showing the real safety and benefit of this treatment.

**Electronic supplementary material:**

The online version of this article (doi:10.1186/1471-2261-15-7) contains supplementary material, which is available to authorized users.

## Background

According to the American Heart Association, noncompaction cardiomyopathy (NCC) is a genetic disorder [1] characterized by intrauterine arrest of the process of myocardial compaction that starts at the 8th week of gestation. Clinical manifestations include ventricular arrhythmia, heart failure, and systemic thromboembolism, especially encephalic. Ventricular hypertrabeculation is believed to be an anatomical substrate for the formation of thrombi. The literature shows an important variation in the incidence of embolic events in NCC between 0% and 38%, with few references to pulmonary thromboembolism [2]. Yousef *et al.* found it in 7% of patients [3]. The main limitation of these findings is the small number of patients in the studies.

## Case presentation

**Figure 1:**
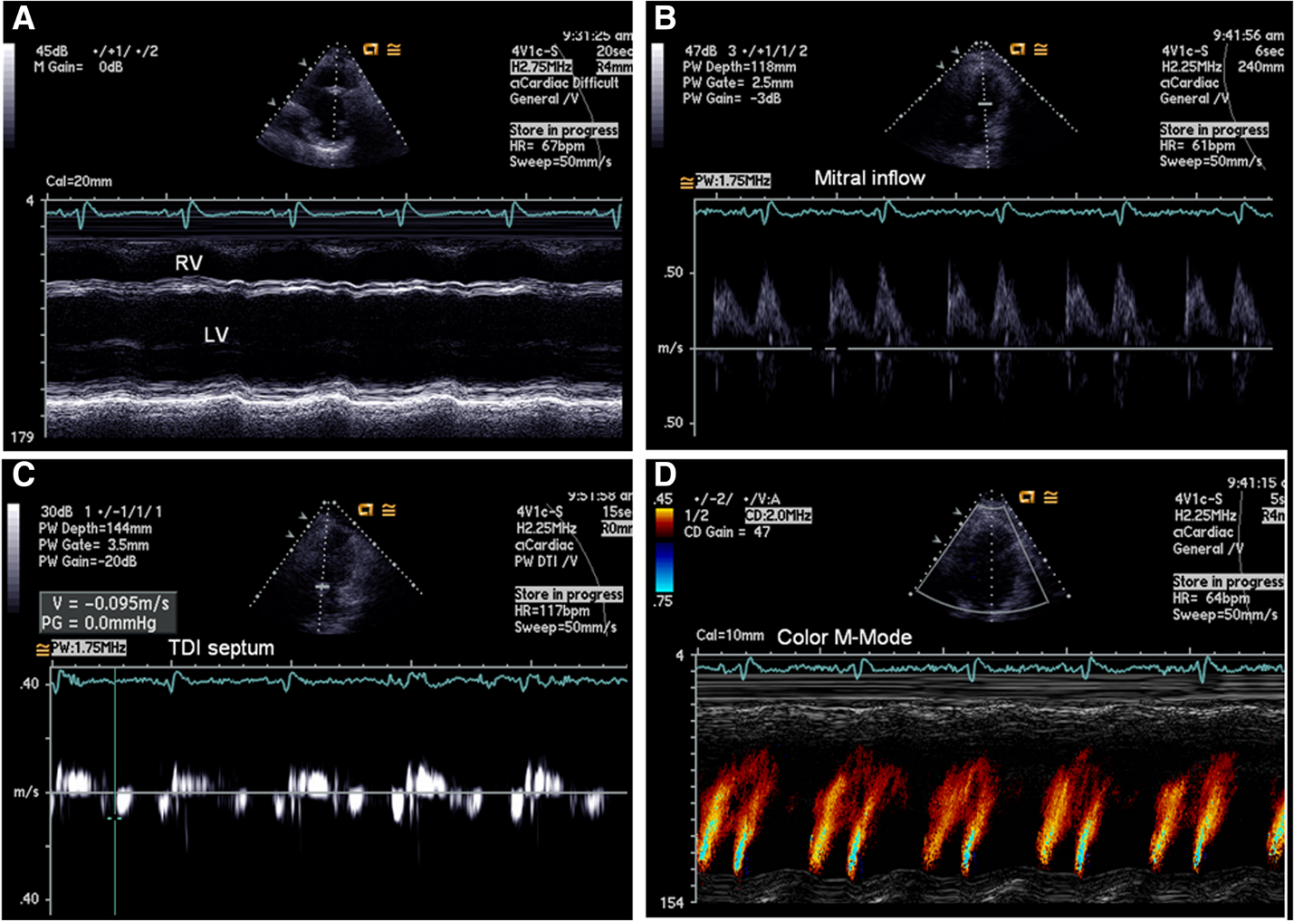
**Echocardiographic images showing severe left ventricular systolic dysfunction (A) and a grade 2 diastolic dysfunction pattern (B to D).**

**Figure 2:**
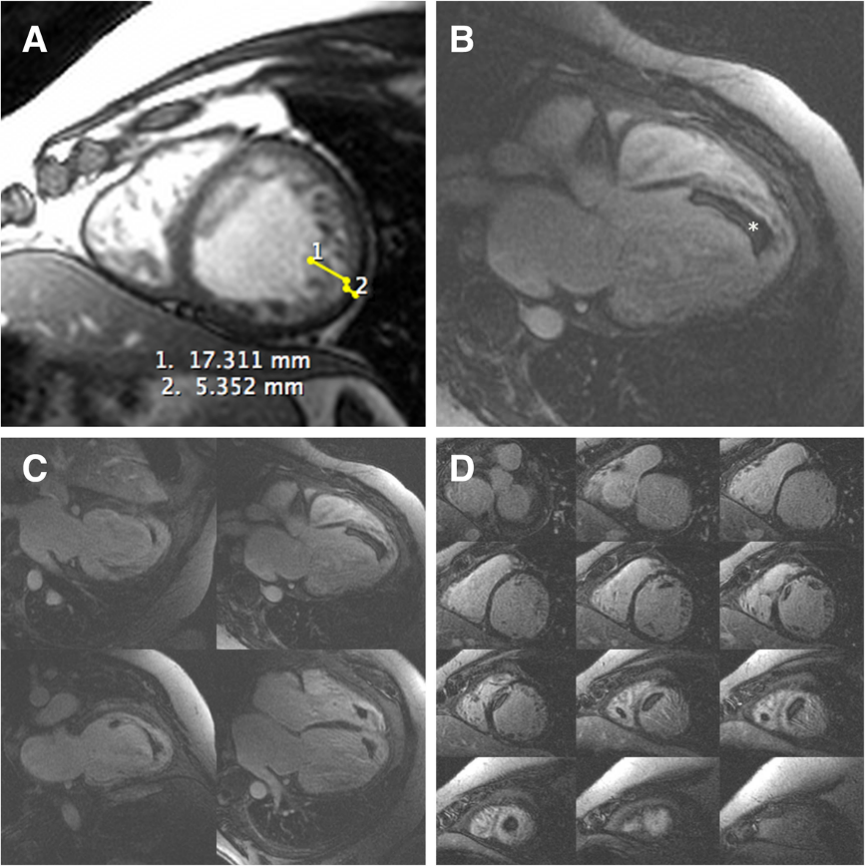
**Cardiovascular magnetic resonance short axis cine images showing left ventricular wall and trabeculation, with maximum non-compacted to compacted thickness ratio of 3.2 (normal < 2.3) (A); delayed enhancement long-axis five-chamber view showing left ventricular apical thrombus (B); multiple long-axis four-chambers disclosing biventricular thrombus (C); multiple short-axis two-chambers illustrating the same biventricular thrombus (D).** LV (left ventricle).

**Figure 3:**
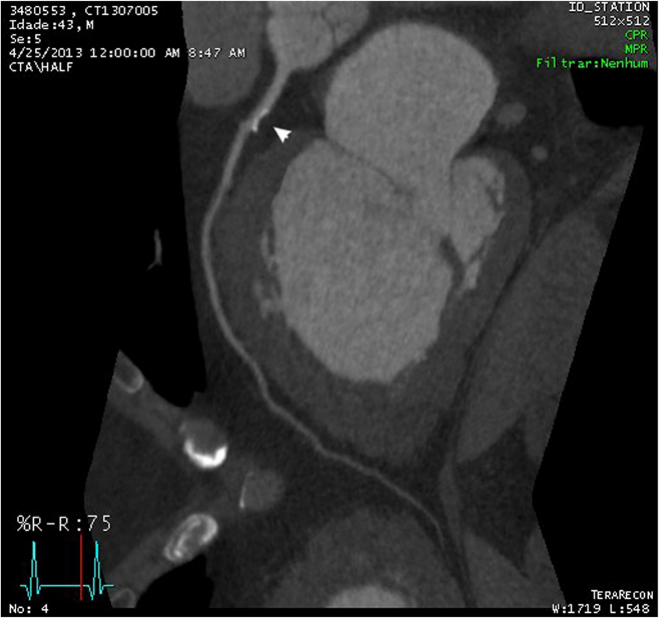
**Coronary computed tomography angiogram showing a calcified nonobstructive plaque in the proximal left anterior descending coronary artery.**

A 43-year-old male, obese, former-smoker patient was suffering from gout and had been anticoagulated by pulmonary thromboembolism for 1 year when he developed progressive dyspnea without anginal complaints. He was directed to a tertiary hospital for investigation of heart failure. Through echocardiographic evaluation, severe biventricular diffuse systolic dysfunction with suspicion of noncompacted cardiomyopathy was identified, even though such a hypothesis could not be confirmed [Figure 1]. Cardiovascular magnetic resonance imaging (CMRI) showed severe biventricular trabeculation with an ejection fraction of 15%, ratio of maximum noncompacted/compacted diastolic myocardial thickness of 3.2 and the presence of exuberant biventricular apical thrombus [Figure 2, Additional file 1]. CMRI disclosed transmural late gadolinium enhancement at 2/17 segments (mid-anteroseptal and apical septal). Coronary computed tomography angiogram showed a calcified nonobstructive plaque in the proximal left anterior descending coronary artery [Figure 3].

In 2008, according to Fazio *et al.*, NCC does not present thromboembolic risk and there is no indication for anticoagulation [4]. On the other hand, nowadays, the indication for anticoagulation treatment in NCC is still debatable. Almeida *et al.* recommended anticoagulation only in cases of left ventricular dilation and dysfunction or with previous embolic events [5]. Recently, Stöllberger and Finsterer stated that thrombi may also develop in patients with NCC even with preserved systolic function [6]. It is generally recommended to those presenting ventricular systolic dysfunction, antecedent of systemic embolism, presence of cardiac thrombus and atrial fibrillation [7]. CMRI plays a crucial role in the diagnosis of left ventricular noncompaction, especially for its accuracy in detecting ventricular thrombi. No prospective study demonstrates the benefits of anticoagulation in NCC patients, which generates uncertainty and insecurity, because there are reports of patients without ventricular dysfunction or atrial fibrillation who have suffered a systemic thromboembolism.

## Conclusion

This case report presents a patient with severe biventricular dysfunction in sinus rhythm, with previous pulmonary thromboembolism and biventricular thrombi. He presents a formal indication for anticoagulation. On the other hand, more studies are necessary to clarify this approach for patients presenting NCC with ventricular dysfunction in sinus rhythm.

## Consent

Written informed consent was obtained from the patient for publication of this Case report and any accompanying images. A copy of the written consent is available for review by the Editor of this journal.

## Electronic supplementary material

Additional file 1: **Four-chamber view cine steady state free precession cardiac MRI.** The left ventricle has increased diastolic volume and severe ventricular dysfunction. (ZIP 4 MB)

## Abbreviations

CMRI: Cardiovascular magnetic resonance imaging
NCC: Noncompaction cardiomyopathy.
